# Homozygous hemoglobin S (HbSS) presenting with bilateral facial nerve palsy: a case report

**DOI:** 10.1186/1756-0500-7-729

**Published:** 2014-10-16

**Authors:** Babatunde Gbolahan Ogundunmade, Unyime Sunday Jasper

**Affiliations:** Department of Physiotherapy, Jos University Teaching Hospital, P.M.B 2076, Jos, Plateau State Nigeria

## Abstract

**Background:**

Bilateral facial nerve palsy is a relatively rare presentation and often points to a serious underlying medical condition. Several studies have reported presentation of bilateral facial nerve palsy in association with Lyme disease, Guillain-Barre syndrome, systemic lupus erythematosus, human immunodeficiency virus, sarcoidosis, diabetes and Hanson disease. While unilateral facial nerve palsy is sometimes associated with hemiplegia in sickle cell patients, no case of bilateral facial nerve palsy have been reported in the literature.

**Case presentation:**

A 29-year-old black African woman who is a known homozygous haemoglobin S (HbSS) presented with bilateral facial nerve palsy. She had the said condition 2 months post delivery of her first child and reported for physiotherapy 3 months post incidence. The pre-treatment House Brackmann Facial Grading Scale (HBFGS) Scores were 3 for right side and 4 for left side. This patient was not on any medication for the facial palsy. After 4 sessions of combination therapy consisting of faradism, facial exercises and massage there was remarkable improvement in the neurological status of the facial muscles. The post treatment House Brackmann Facial Grading Scale score was 2 bilaterally.

**Conclusion:**

Bilateral facial nerve palsy may be an initial presentation of sickle cell anemia patients in the absence of other overt clinical presentations. Therefore sickle cell anemia should be considered among others, in the differential diagnosis of bilateral facial nerve palsy. Furthermore, this case report has highlighted the important role of physiotherapy in the management of bilateral facial nerve palsy.

## Background

Injury to the seventh cranial nerve may result in a condition known as facial palsy. This involves the paralysis of the muscles of facial expression. There are two distinct possible facial nerve lesions: peripheral and central facial nerve lesions. The lesion site differs in the two instances. Motor neurons in the brainstem nucleus are damaged in peripheral palsy while the lesion site in central palsy is located central to the supranuclear lesion. A central lesion (upper motor neuron lesion) such as hemispheric stroke preserves forehead wrinkling and cause only mild weakness of eye closure, while the lower face is more severely involved [1]. If there is a peripheral lesion (lower motor neuron lesion), the eyelids will gape open. Some cranial neuropathies or neuromuscular diseases cause facial weakness. Paralysis of the facial muscles can have a significant impact on a patient’s physical, social and psychological status [2]. Physically, patients with facial nerve palsy can present with facial asymmetries and motor difficulties (eating, drinking and speaking). Socially, there is a challenge of conveying emotions through facial expressions (happiness, sadness and surprise). The psychological effect is seen in the devastating impact on a person’s self esteem.

Bilateral facial nerve palsy unlike its unilateral equivalent which is more generic, is an exceptionally rare clinical presentation. While unilateral facial paralysis has an incidence of 20–25 per 100,000 population, bilateral facial palsy or facial diplegia occurs in 0.3–2% of facial paralysis patients with an annual incidence of approximately 1 per 5 million [3]. Furthermore, while most unilateral facial palsy is idiopathic, with bilateral facial palsy an aetiological factor is often demonstrable [4]. The majority of these cases are due to serious underlying medical conditions such as Lyme disease, Guillain-Barre syndrome, leukaemia, sarcoidosis, infectious mononucleosis and trauma and may need emergency medical treatment. Only 20% of these cases are due to idiopathic or Bell’s palsy where no evidence of systemic or local disease can be found [3].

In homozygous haemoglobin S (HbSS), a valine for glutamic acid substitution occurs on both ß-globin chains because of the inheritance of mutated ß-globin chain genes from both parents. The condition is described as “sickle cell anaemia or sickle cell disease” because of the sickle shaped red blood cells that occur when there is a “sickle cell crisis”. The most common neurological complication associated with sickle cell anaemia is hemiplegia which is often accompanied by unilateral facial nerve palsy. After performing a thorough literature search, we found no reports of homozygous haemoglobin S (HbSS), sickle cell anaemia or sickle cell disease causing bilateral lower motor neuron facial palsy. To the best of our knowledge this is the first case of bilateral facial palsy associated with HbSS to be reported. However, while some of the case reports of bilateral facial palsy in literature have been successfully treated with medication, the patient presented in this report was not placed on any medication at the time she was referred for physiotherapy management.

## Case presentation

The patient was a 29-year-old Black African woman referred for physiotherapy with a diagnosis of bilateral facial palsy 3 months post incidence. There was history of sudden onset of the condition when she woke up from her sleep. She was a known sickle cell patient and had her first delivery of a baby girl 2 months pre-incident of the condition. The delivery was by caesarean section done under spinal anaesthesia after 9 months gestation period. The patient was not diabetic nor hypertensive. There was no history of ear infection, influenza or trauma to the head/temporal region. There was no antecedent headache, recent travel or previous history of facial nerve palsy. She however had sickle cell crises 9 weeks post incidence of the condition.

There was no history of trauma and travel in the 5 weeks preceding onset of condition. There was no history of smoking, alcohol intake, unsupervised or unprescribed blood transfusion, or engaging in promiscuous sexual activities. Power in all limbs was normal; deep tendon reflexes were normal. Sensory examination was unremarkable and there were no cerebellar signs.

Medical examinations and report had earlier ruled out any other associated conditions such as viral infections, metabolic, vasculitic and immunological conditions. All other cranial nerves were intact; her motor system examination was normal; she had a normal gait and speech and co-ordination was normal. Affectation of other cranial nerves was also ruled out as were other neurological signs and conditions. She was seronegative for human immunodeficiency virus (HIV).

### Investigations

Full blood count, urea and electrolytes, bone profile, liver function tests and glucose were all within normal limits. Post incidence magnetic resonance imaging (MRI) of the brain revealed a subarachnoid cyst.

### Initial physiotherapy assessment

Examination at presentation revealed:

- Facial grimacing
- Inability to wrinkle forehead skin
- Bilateral loss of nasal folds
- Inability to smile, laugh and talk properly
- Inability to whistle and close both eyes (positive Bells phenomenon).
- No swelling/tenderness/pain at the parotid gland region
- Severe synkinesis on left side and slight synkinesis on right side
- House Brackmann Facial Grading Scale (HBFGS) [5] Score: Right = 3, Left = 4
- Gross Muscle Power = 5, bilaterally (oxford muscle grading).

### Treatment procedure

Patient was treated on alternate days with the following treatment regime:

1. Faradic Stimulation from Enraf Nonius Sonoplus 6 series was applied to both sides of the face with the following treatment parameters:
  Triangular wave
  Phase duration = 1 second
  Phase interval = 300 milliseconds
  Current intensity = 9.2 mA
  Time = 10 minutes.
2. Facial Exercises, 20 repetitions of assisted active exercises to each of the facial muscles using mirror as biofeedback.
3. Soothing stroking massage (20 repetitions) with talcum powder to both sides of the face.
4. Treatments 2 and 3 were given as home programme to be done thrice daily (this was confirmed from the patient whenever she reported for treatment).

### Outcome of physiotherapy management

After 4 treatment sessions of the above stated therapy, patient had to relocate. The assessments at this point are as follows:

- Slight asymmetry of smile with maximal effort
- Synkinesis was barely noticeable
- Complete closure of the eye
- Restoration of nasal folds
- HBFGS Score was 2 bilaterally.

## Discussion

The most common neurological complication associated with sickle cell anaemia is hemiplegia which is often times accompanied by facial nerve palsy. However, there is no mention of bilateral facial nerve palsy as a neurological impediment in sickle cell anemia (SCA) either in isolation or in company with stroke found in literature. Earlier a study reported that anaesthesia administered to a sickler precipitated the appearance of unexpected and bizarre neurological findings [6]. While the aforementioned study did not outline what the “bizarre” findings were, the patient in this study received spinal anaesthesia during child delivery which may have triggered the neurological presentation of bilateral facial nerve palsy. Nerve damage which may be temporary or permanent is a rare complication of spinal anaesthesia [7]. Basic and clinical evidence has shown that local anaesthesia may cause nerve injury either by direct toxicity to the axon or Schwann cell or by secondary disruption of the nerve microenvironment [8]. The effects of local anaesthesia on nerve blood flow may be related to inhibition of endothelium-dependent vasodilation or the synthesis of vasodilating prostaglandins [8]. Nimala and Kumari [9] have reported a case of foot drop following spinal anaesthesia in a 28-year-old woman.

Furthermore infarction and small haemorrhages are some of the pathophysiological sequela of sickle cell crisis which results in varied neurological presentation, of which bilateral facial nerve palsy may be one. While it can be argued that the sickle cell crisis was not primarily associated with her presentation since she only had a sickle cell crisis 9 weeks post presentation, it may be that the neurologic symptom of bilateral facial nerve palsy is a potential sign of ongoing or evolving cerebrovascular disease [6]. Also the peculiar nature of their white and red blood cells such as high steady-state neutrophil count and excessive adhesion of white blood cells to vessel endothelium predisposes them to neurological symptoms such as stroke even when they are apparently healthy [6]. It therefore portends the consideration of haemoglobinopathies such as SCA while probing for potential causes of bilateral facial palsy even among apparently healthy sufferers.

Wherefore metabolic, infectious, vasculitic, traumatic, immunological (e.g., multiple sclerosis) and neoplastic causes should be considered before diagnosing a bilateral idiopathic or Bell’s palsy; it should also be noted that even though the commonly reported neurologic complication for SCA is overt stroke, a high frequency of silent infarction is revealed by neuroimaging studies in asymptomatic SCA patients [6]. Therefore, it is plausible that bilateral facial palsies may be among the inexplicable consequences of this quiet change in the brain of SCA patients in the absence of other overt clinical presentations. Furthermore common causes for bilateral facial palsy include Lyme disease, Guillain-Barre syndrome, Leukaemia, sarcoidosis, infectious mononucleosis and trauma. Medical examination of our patient ruled out of the above conditions. Therefore, sickle cell disease should be considered as part of the differential diagnosis of bilateral nerve palsy in addition to the aforementioned conditions.

Tissues of the central nervous system are very sensitive to the most temporary hypoxia and sickle cell disease being systemic in nature predisposes sufferers to diverse neurological symptoms. These include variable disturbance of reflexes, facial weakness, nystagmus, transitory blindness and homonymous. While it may not be sufficient to conclude that the presentation of bilateral facial resulted from the subarachnoid cyst seen post incidence on MRI, one of the diagnosis considered for the numerous neurological defects seen in sickle cell patients was subarachnoid haemorrhage [6]. Furthermore, the patient in this study did not present with any of the typical symptoms of subarachnoid cyst such as headache, nausea and vomiting, seizures, hearing and visual disturbances, vertigo, and difficulties with balance and walking [10].

Even though one study which reported bilateral facial nerve palsy in Guillan Barre indicated the utilization of intensive physiotherapy to aid in the recovery process [11], it did not state the physical therapy modality employed as part of the management. The findings of this report suggests that in addition to the medical treatment of bilateral facial nerve palsy, physiotherapy management should be included as a core part of the treatment protocol especially at the acute stage. It is plausible that for those with lingering weakness after medical treatment, intensive and extensive physiotherapy management may be the only means of ameliorating any residual functional impairment. Besides, these may help speed up and maintain the recovery process. For example, while it took 30 days for the GBS patient with bilateral facial palsy to recover completely on the right with residual deficit on the left (House-Brackmann = II; same as our study) with medical treatment [11], the patient in our report recovered in two-weeks. This is even more so as there are very minimal or no side effects associated with the use of physiotherapy-related modalities in the management of bilateral facial nerve palsy as may be obtained with medications.

While literature has revealed that recovery usually begins within 2–4 weeks reaching its maximum within 6–12 months [12], our patient was still at baseline House Brackmann score when she was referred for physiotherapy 3 months post incidence. Furthermore at the time of discharge, the patients House Brackmann score was 2 bilaterally a finding which is at variance with the claim that one side of the face recovers faster than the other in bilateral facial nerve palsy [13]. This is probably due to differences in the conditions and management approach. While faradic stimulation and other physical therapy treatment methods were employed in managing this patient, the others did not.

The improvement noticed in this patient’s condition is supported by a study carried out by Lindsay *et al*. [14] which reported statistically significant increases in Facial Grading Scores after treatment (p < 0.001, t-test) with faradic stimulation and facial exercises. The use of faradic stimulation enhances activation and maintenance of healthy motor units of fully and partially innervated muscles. This brings about preservation and rejuvenation of the physiological properties of the facial muscles and prevention of disuse atrophy while function is still impaired, thereby reducing disfiguring facial asymmetry. It thus shows that Physical therapy can help reduce facial disability and ensure social re-integration of the patient with reduced psychological effects [15]. Targen *et al*. [16] demonstrated positive effects of long term electrical stimulation on motor recovery in patients with unresolved facial nerve palsy.

The common belief that there is natural tendency for spontaneous recovery from bell’s palsy should not hamper referral of facial palsy patients for Physical therapy; this is due to possible deterioration of motor function in the recovery period however marginal this period may be. Increasing evidence shows that the way the patient is managed has an important effect on outcome. Untreated facial palsy leaves some patients with major facial dysfunction and a reduced quality of life [17]. Improving outcomes requires effective collaboration between physiotherapists, ophthalmologists and general practitioners The treatment modalities used in this report may as well be used in isolation to demonstrate the possible effectiveness or otherwise of the use of single modality therapy in the management of facial palsy.

## Conclusion

There is no report of bilateral facial nerve palsy in a sickle cell patient in literature. The presentation of bilateral facial nerve palsy may be attributed to either the spinal anesthesia she received during labour or the silent neurological changes in the patients CNS due to SCA. This report has highlighted a need to include heamoglobinopathies such as SCA in the differential diagnosis of bilateral facial nerve palsy. Furthermore, the important role Physical therapy plays in the management of facial palsies whether unilateral or bilateral has also been established.

## Consent

Written informed consent was obtained from the patient for publication of this case report. A copy of the written consent is available for review by the Editor-in-Chief of this journal.
